# Unveiling the Uncommon Ventriculitis in Tubercular Meningitis

**DOI:** 10.7759/cureus.89312

**Published:** 2025-08-04

**Authors:** Garima Misra, Sree Nithya Kosaraju, Bhawarpreet Singh Bhatti

**Affiliations:** 1 Neurology, ESIC Medical College and Hospital, Hyderabad, IND; 2 Neurology, Raja Rajeshwari Institute of Medical Sciences, Hyderabad, IND; 3 Neurology, Government Medical College, Amritsar, Amritsar, IND

**Keywords:** case report, central nervous system tuberculosis, mri diagnosis, tubercular meningitis, ventriculitis

## Abstract

Tubercular (TB) meningitis is one of the manifestations of central nervous system tuberculosis, a form of extrapulmonary tuberculosis. Despite the high incidence of TB meningitis in developing countries, there are hardly any reports of associated ventriculitis, making it one of the rare complications. Ventriculitis complicating TB meningitis is devastating not only to the immunocompromised but also to the immunocompetent population. The diagnosis of TB meningitis is indeed challenging, owing to the clinical similarities with other types of meningitis and laboratory techniques that are rather insensitive and slow. Thus, this under-recognized complication can impact the morbidity and mortality of the people affected by it, making it imperative for it to be diagnosed and managed early.

We present a case of a 52-year-old man with no known comorbidities, who presented with fever, chills, headache, vomiting, and altered mental status for four days, and showed a Glasgow Coma Scale (GCS) score of 8 (E2V2M4), stiffness of the neck, sluggishly reactive pupils, and tachycardia on arrival. The pathological findings, including CSF analysis, MRI, and cartridge-based nucleic acid amplification test (CBNAAT), diagnosed the case as TB ventriculitis with meningitis. The patient was mechanically ventilated and then treated with anti-TB treatment and steroids.

The case thus illustrates a rare and challenging presentation of TB meningitis that can present with a variety of neurological sequelae and complications, including ventriculitis, as in this case, which can have devastating consequences if left untreated. It can result in persistent neurological sequelae, hydrocephalus, and prolonged hospital stay. Hence, our case highlights the need for a timely diagnosis and treatment that can help improve the prognosis, thereby reducing morbidity and mortality.

## Introduction

Central nervous system tuberculosis is an uncommon but life-threatening form of tuberculosis that is associated with high mortality and significant neurological morbidity. The most commonly reported manifestations include tubercular (TB) meningitis, intracranial tuberculomas, and pyogenic ventriculitis. Globally, central nervous system tuberculosis has been found to comprise approximately 13.9% of all meningitis cases and 4.6% of all tuberculosis cases, as reported in a systematic review and meta-analysis assessing its incidence and prevalence across various clinical settings. Based on the literature review, only four cases of TB pyogenic ventriculitis have been reported in the literature until 2024 [1-3]. 

The case detection ratio (CDR) for TB meningitis amongst adults in 2019 was 164,000 (95% UI; 129,000-199,000) globally [2]. Even in India, a country with a high tuberculosis burden (tuberculosis case rate of 179 per 100,000 population) and numerous cases of TB meningitis (5-7% of all the admissions in neurology and pediatric wards in super specialty hospitals), ventriculitis remains a poorly recognized complication [4].

The mechanism behind ventriculitis in tuberculosis could be attributed to a hematogenous spread of infection via the choroid plexus, even without having pulmonary tuberculosis. In cases where it is associated with pulmonary tuberculosis, the tubercle bacilli are inhaled via droplets, infecting the lung and lymph nodes initially, and thereby spreading through the bloodstream to other areas. This, in some circumstances, would lead to an encapsulated pus collection within the ventricular system, also called ventriculitis [1,2]. TB meningitis presents with a variety of features like altered mental status, focal neurological deficits, seizures, cranial nerve palsies, and meningitis-like symptoms. Likewise, TB ventriculitis could present with headache, dizziness, loss of consciousness, and even death sometimes. Other complications include persistent neurological deficits, hydrocephalus and tuberculomas [5,6]. 

Apart from the clinical perspective, a CSF examination is crucial for an early and reliable diagnosis of TB meningitis. Typical changes include lymphocytic pleocytosis, decreased glucose levels, and elevated protein levels. The gold standard for microbiological confirmation is the demonstration of *M. tuberculosis* in the CSF. It can also become a tool in delineating between pyogenic, TB, and fungal meningitis with the help of CSF Gram stain and culture, CSF cartridge-based nucleic acid amplification test (CBNAAT) (or CSF adenosine deaminase (ADA)) and CSF fungal rapid antigen test, respectively [7]. In addition to this, neuroimaging via MRI is also considered valuable for detecting abnormalities due to TB meningitis. The usual imaging findings include leptomeningeal and basal cisternal enhancement, ventriculomegaly due to hydrocephalus, periventricular infarcts, and the presence of tuberculomas. Ventriculitis as a complication in TB meningitis shows ependymal enhancement, dilated ventricles, and debris with irregular margins in the dependent portions of the ventricles on imaging [8]. Combining both presentations is seen as a rare complication, and if left undetected, could lead to catastrophic consequences.

## Case presentation

A 52-year-old man with no known comorbidities presented to the emergency department with complaints of fever, chills, vomiting, and headache associated with altered mental status for four days. Upon arrival, his Glasgow Coma Scale (GCS) score was 8 (E2V2M4), he was tachycardic (110 beats per minute), and febrile (38.3°C). His initial evaluation revealed stiffness in the neck, along with pupils that were sluggishly reactive to light.

The laboratory tests conducted on the first day of hospital admission demonstrated an elevated total leukocyte count (27,300/mm^3^), a raised erythrocyte sedimentation rate (ESR), and no gross abnormalities on non-contrast CT of the brain.

The results of initial laboratory investigations are listed in Table 1.

**Table 1:** Results of initial laboratory investigations CBC: Complete blood count; ESR: Erythrocyte sedimentation rate; HbsAg: Hepatitis B surface antigen

Investigations	Patient's Findings	Normal Values
CBC		
Hemoglobin	14.4 gm/dL	8.90-11.90 gm/dL
White blood cells	27,300/mm^3^	6700-14,200/mm^3^
Platelets	2.5 mm^3^	2.75-5.67 mm^3^
Serum electrolytes		
Sodium (serum)	136 mmol/L	136-146 mmol/L
Potassium (serum)	3.7 mmol/L	3.50-5.00 mmol/L
Renal function test		
Serum creatinine	0.7 mg/dL	0.30-1.20 mg/dL
Blood urea	27 mg/dL	15.00-45.00 mg/dL
ESR	85 mm/hr	1.00-10.00
Procalcitonin	11.9 ng/mL	0.00-0.50 ng/mL
Viral markers		
HbsAg	Negative	Negative
Antibodies for HIV 1, and 2	Non-reactive	Negative
Hepatitis C antibody (rapid)	Non-reactive	Negative

On the second day of hospital admission, the patient underwent a repeat leukocyte count level, CSF analysis and tuberculin skin test (TST). The CSF analysis for the patient revealed a total cell count of 200 with 93% lymphocytes, and 7% neutrophils, CSF protein 320 mg/dL, CSF sugar of 38 mg/dL, ADA levels of 23.27 IU/L, and a positive CSF CBNAAT result. The CSF Gram stain and culture showed no bacterial growth, thus ruling out the possibility of pyogenic TB meningitis and ventriculitis. Similarly, the possibility of a fungal etiology was ruled out on the basis of negative CSF fungal antigen rapid test. The results of these diagnostic investigations (Day 2) are represented in Table 2.

**Table 2:** Results of subsequent diagnostic investigations TLC: Total leukocyte count; ESR: Erythrocyte sedimentation rate; TST: Tuberculin skin test; TB: Tubercular; ADA: Adenosine deaminase; CBNAAT: Cartridge-based nucleic acid amplification test

Parameter	Patient Value	Units	Reference Range	Interpretation	Comments
TLC	25,300	/mm³	4,000 – 11,000	High (leukocytosis)	Suggests systemic infection
ESR	85	mm/hr	<20 (male)	High	Non-specific inflammatory marker
TST	Positive	–	Negative	Positive	Tuberculosis exposure likely
CSF total cell count	200	cells/mm³	0-5	High (pleocytosis)	Indicates central nervous system infection
CSF differential count	93% lymphocytes, 7% neutrophils	%	Lymphocyte-predominant expected in tuberculosis	Lymphocytic pleocytosis	Consistent with TB meningitis
CSF protein	320	mg/dL	15-45	High	Protein elevation common in tuberculosis
CSF glucose	38	mg/dL	45-80 or ≥2/3 of serum glucose	Low	Hypoglycorrhachia seen in TB meningitis
CSF ADA	23.27	IU/L	<10 (typically)	High	Suggestive of central nervous system tuberculosis [7]
CSF CBNAAT	Positive	–	Negative	Positive	Confirmatory for *M. tuberculosis*
CSF Gram stain and culture	No bacterial growth		–	No bacterial growth	Rules out pyogenic meningitis and ventriculitis
CSF fungal antigen rapid test	Negative for Cryptococcal antigen		–	Negative for Cryptococcal antigen	Rules out fungal meningitis and ventriculitis

Also, on the second day of admission, the patient underwent a chest X-ray that revealed lower zone opacification in the left lung. Following this, he underwent a high-resolution CT scan of the chest that showed few subcentrimeric lymph nodes in the prebrachial region, along with fibrotic bands in the left posterobasal segment.

**Figure 1:**
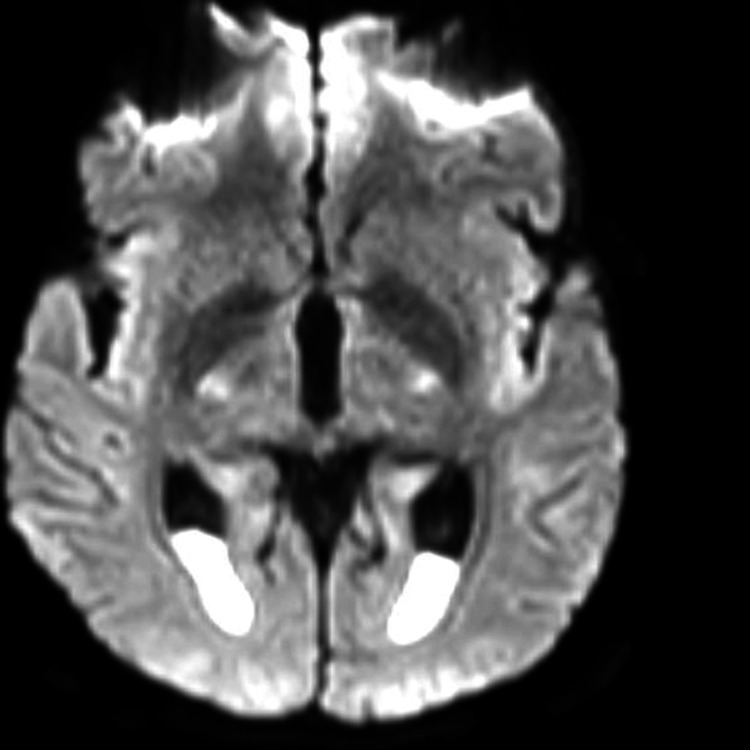
Axial DWI reveals hyperintense signal in the bilateral temporal and parieto-occipital lobes suggestive of restricted diffusion DWI: Diffusion weighted imaging

**Figure 2:**
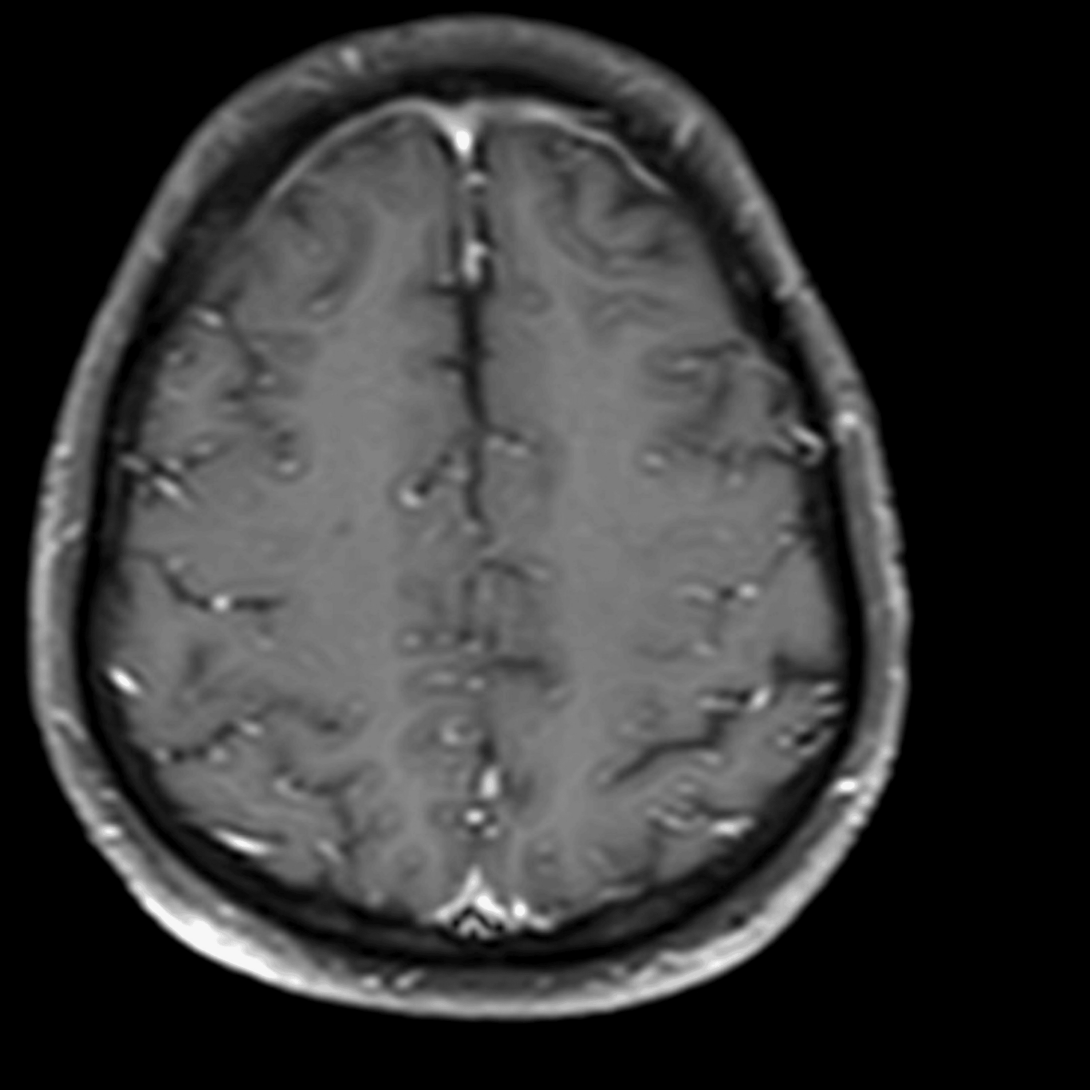
Post-contrast T1-weighted axial image demonstrates subtle leptomeningeal enhancement, particularly in the cortical sulci of the frontal lobes, suggestive of meningeal inflammation

**Figure 3:**
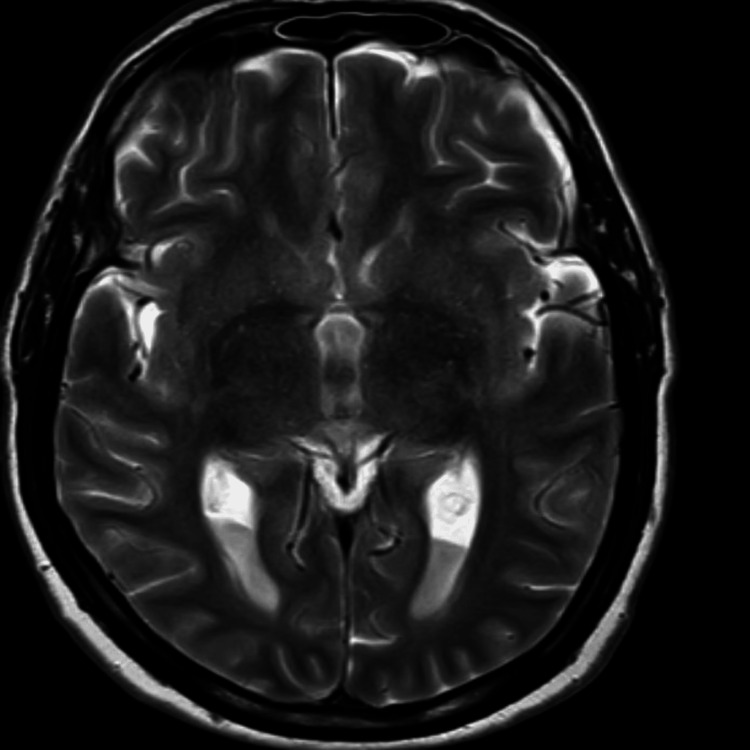
T2-weighted axial image at the level of the thalami demonstrates bilateral symmetrical hyperintensity involving the thalamus and posterior limb of internal capsule, with surrounding white matter also appearing mildly hyperintense

**Figure 4:**
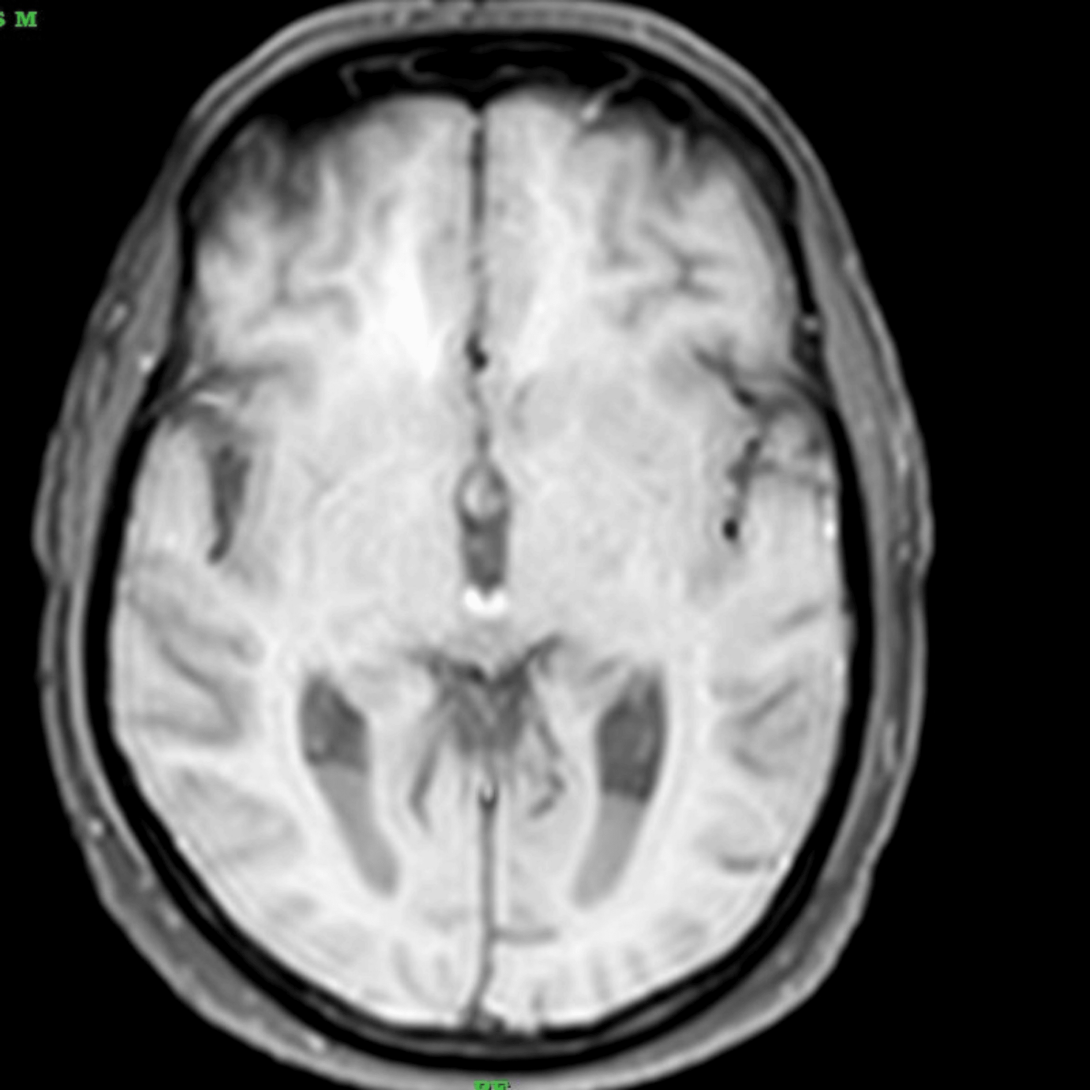
Axial T1-weighted MRI image shows isointense basal ganglia structures with relatively hypointense CSF, consistent with T1 characteristics

**Figure 5:**
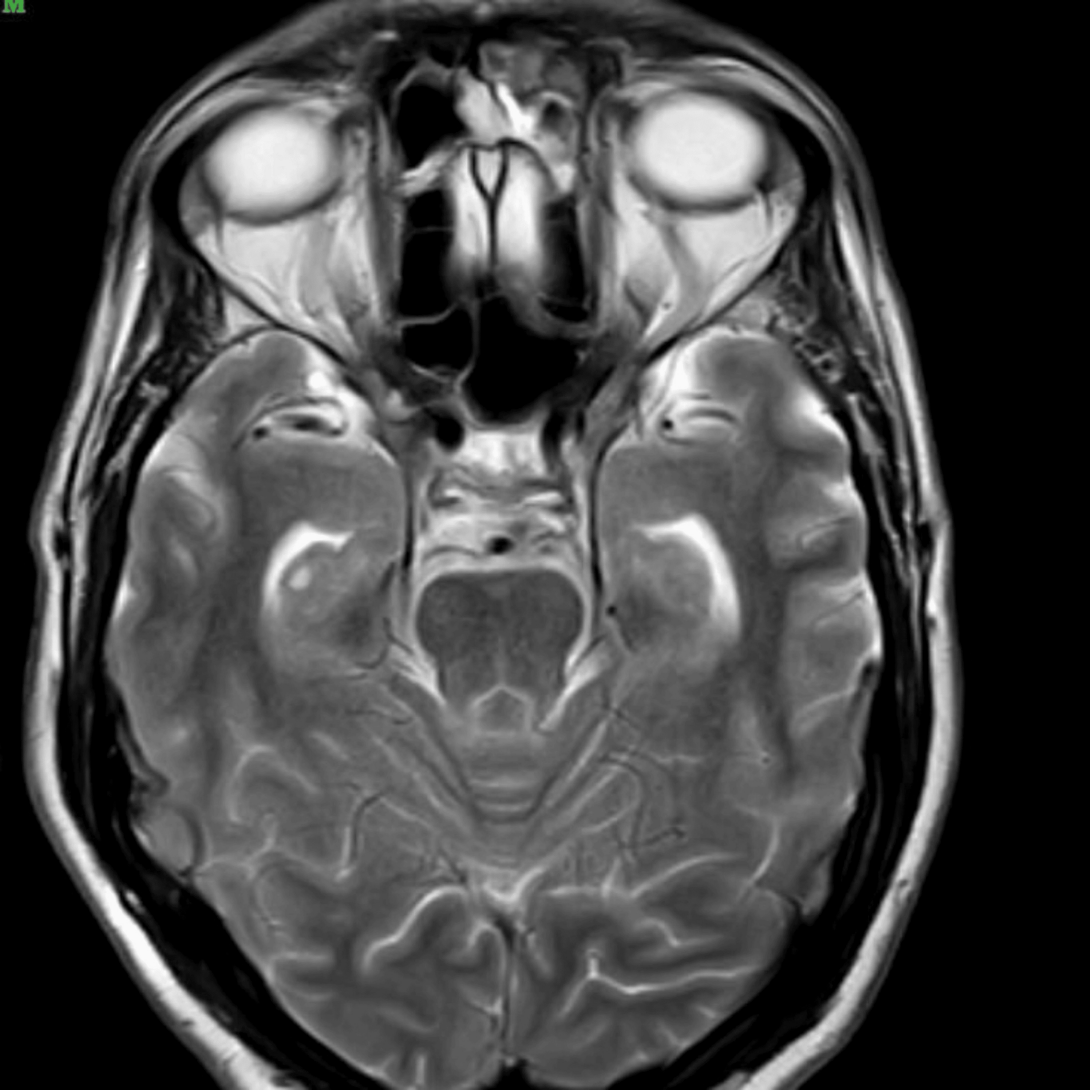
Axial T2-weighted MRI demonstrates hyperintense signal changes in the bilateral mesial temporal lobes and basal ganglia, with prominent CSF spaces

 On the same day of the hospital stay, a contrast MRI of the brain was performed. This scan revealed a fluid-fluid level with layering in the occipital horns of the bilateral lateral ventricles, showing restriction on diffusion weighted imaging (DWI)-exudates, and anterior dural and mild meningeal enhancement. In addition, it also showed a few T2/fluid attenuated inversion recovery (FLAIR) hyperintensities in the periventricular white matter with mildly dilated and tortuous bilateral superior ophthalmic veins. All these findings were suggestive of meningitis with ventriculitis. The radiological findings described above are depicted in Figures 1-5.

Management

The patient had a hospital stay of total 14 days, including an initial stay in the ICU, followed by seven days in the neurology inpatient unit. He was treated by medical therapy alone, as the surgical management is offered only to those patients who develop hydrocephalus or show failure to improve with medical management. 

Considering his clinical symptoms, elevated leukocytes, and raised ESR levels, he was prescribed empirical antibiotics from the first day of admission. This included injection ceftriaxone (2 g twice daily intravenously) for four days, and injection vancomycin (1 g twice daily intravenously) for 14 days. On the fifth day, injection ceftriaxone was stopped, and the patient was prescribed injection meropenem (1 g thrice daily) for the next 10 days due to persistently elevated leukocyte counts. 

Owing to the radiological and laboratory evidence for TB meningitis with ventriculitis, he was administered anti-TB drugs and steroids for the same, beginning from the third day of admission. The anti-TB drug regimen was: tablet isoniazid (H) 5 mg/kg (300 mg); tablet rifampicin (R) 10 mg/kg (600 mg); tablet pyrazinamide (Z) 25 mg/kg (1,500 mg); tablet ethambutol (E) 15 mg/kg (900 mg) for two months, followed by the drugs HRE for the next 7-10 months. He was also advised to take tablet pyridoxine 40 mg along with this regimen.

Likewise, the steroid regimen was started on Day 3 with injection dexamethasone 24 mg/day for the first week, followed by 16 mg/day for the next week, and then gradually tapered and stopped. So, the patient received six to eight weeks of steroid treatment which was shifted from intravenous route (dexamethasone) to oral (prednisolone) route after discharge. 

The patient also underwent intubation and was placed on mechanical ventilation on the third day of admission on account of declining GCS (E1V2M4), respiratory distress, and desaturation. Regular monitoring of the vital signs, and care of endotracheal tube was done daily. He was extubated on the seventh day of admission, when clinical improvement (GCS E4VTM6) and stable vital signs (no signs of desaturation or tachypnea) were observed. He still received oxygen support for the next two days and weaned off the support later. 

On the seventh day of admission, the leukocyte counts had normalized to 8,900/mm^3^ and fever had subsided. He also underwent a repeat CT scan of the brain on the 12th day of admission, which showed no new complications. Considering all these factors, along with improvement in his clinical status, vital signs, and decreased need for oxygen support, he was discharged on the 14th day of admission in a hemodynamically stable condition. On follow up after two months, he had completed his steroid treatment and was still undergoing anti-TB treatment. He denied any fresh complaints and did not develop any persistent neurological deficits or organ dysfunction. 

## Discussion

TB ventriculitis, though uncommon, is a rarely recognized complication of TB meningitis with potentially serious outcomes. It represents inflammation of the ventricular system, often manifesting with ependymal enhancement, intraventricular debris, and hydrocephalus. Despite its severity, it remains underdiagnosed due to its nonspecific clinical presentation and limited awareness in routine practice [1].

In our case, the patient presented with altered mental status and characteristic imaging findings, including layering of intraventricular fluid and smooth ependymal enhancement, features that align closely with those described by Singh et al. in their retrospective analysis of TB ventriculitis cases [1]. Notably, the presence of intraventricular debris with restricted diffusion supports a diagnosis of ventriculitis with purulent content, as emphasized by Obame et al [2]. These findings suggest active inflammation and possible necrosis within the ventricular lining.

The proposed mechanisms of ventricular involvement in tuberculosis include hematogenous dissemination through the choroid plexus and direct rupture of subependymal tubercles into the ventricular cavity [1]. In our case, the inflammation around the choroid plexus supports the former mechanism, consistent with what has been described in prior literature. Recognizing these mechanisms is important, as they can influence both the pattern of spread and the severity of clinical presentation.

One of the challenges in diagnosing TB ventriculitis lies in its imaging overlap with pyogenic infections. However, in tuberculosis-endemic regions like ours, the radiological presence of ventricular debris, smooth ependymal enhancement, and magnetization transfer (MT) hyperintensities should prompt consideration of tubercular etiology [8,9]. While intraventricular debris and ventricular wall enhancement are common MRI findings in all types of ventriculitis, certain features help distinguish tubercular from pyogenic causes. Pyogenic ventriculitis often shows irregular fluid levels and dense pus, whereas tubercular etiology is more likely to present with intraventricular septations, sequestered ventricles, and a hyperintense ependymal wall on MT imaging [4]. Our patient’s MRI showed these characteristic signs (intraventricular exudates with layering in the occipital horns, diffusion restriction, anterior meningeal enhancement, and periventricular white matter hyperintensities - findings suggestive of meningitis with ventriculitis), strengthening clinical suspicion and guiding early treatment initiation [10]. Apart from radiological imaging, even CSF analysis, including Gram stain, CBNAAT, and fungal rapid antigen test, can help distinguish between pyogenic, TB, and fungal causes behind meningitis with ventriculitis. 

Management of TB ventriculitis centers on the early and aggressive initiation of anti-TB therapy, along with adjunctive corticosteroids. Our patient was treated without surgical intervention and demonstrated gradual neurological improvement, consistent with reports that highlight favorable outcomes with medical therapy alone in select cases [8,9]. However, timely diagnosis is crucial, as untreated or late-recognized ventriculitis may lead to complications such as obstructive hydrocephalus, brain abscesses, or even rupture of intraventricular tuberculomas, as illustrated in other reports [11-13].

Overall, this case contributes to the existing literature by elaborating about a case of an immunocompetent host with TB meningitis with ventriculitis, with rare imaging findings (only prior four cases described), who was managed conservatively (medical therapy alone). It further reinforces the importance of recognizing ventriculitis as a distinct entity within the TB meningitis spectrum. Clinicians must be attuned to its subtle clinical cues and characteristic radiological signs, especially in tuberculosis-endemic settings. It should be considered in the patients having TB meningitis presenting with imaging showing ventricular debris, even if pulmonary tuberculosis is absent. Early initiation of appropriate therapy, before irreversible neurological damage occurs, can significantly improve patient outcomes. 

## Conclusions

TB meningitis has a moderately high mortality rate and can lead to residual deficits and a myriad of neurological sequelae if left untreated. The prognosis is worse if the patient develops ventriculitis as a rare complication. A prudent analysis identifying the clinical symptoms, signs, laboratory values, and imaging findings can contribute to an accurate diagnosis in this case. Another inference that can be drawn from this case is the positive effect of early management that can improve the outcomes.

This case report highlights a case of TB meningitis with ventriculitis in an immunocompetent host with rare imaging findings, who was treated by medical therapy alone, and showed subsequent improvement. This case further emphasizes the need for potential improvements in diagnostic modalities and advancements in treatment protocols in the years to come. Altogether, it underscores the importance of a high clinical suspicion, early imaging, and timely initiation of therapy. 

Future directions may include the development of improved imaging-based diagnostic algorithms, incorporation of such findings into TB meningitis diagnostic criteria, and validation through prospective studies.
